# 5,5′-Di-4-pyridyl-2,2′-(*p*-phenyl­ene)di-1,3,4-oxadiazole

**DOI:** 10.1107/S1600536809050557

**Published:** 2009-11-28

**Authors:** Rui-Sha Zhou, Jiang-Feng Song

**Affiliations:** a Department of Chemistry, College of Science, North University of China, Taiyuan, Shanxi 030051, People’s Republic of China

## Abstract

In the crystal structure of the title compound, C_20_H_12_N_6_O_2_, the mol­ecules are located on centres of inversion. The complete mol­ecule is almost planar, with a maximum deviation from the mean plane of 0.0657 (1) Å for the O atom. In the crystal, mol­ecules are stacked into columns elongated in the *a* axis direction. The centroid–centroid distances between the aromatic rings of the mol­ecules within the columns are 3.6406 (1) and 3.6287 (2) Å. Mol­ecules are additionally connected *via* weak inter­molecular C—H⋯N hydrogen bonding.

## Related literature

For the potential uses of oxadiazo­les, see: Bentiss *et al.* (2000 ▶); Navidpour *et al.* (2006 ▶). For related studies on oxadiazo­les, see: Wang *et al.* (2005 ▶); Zhang *et al.* (2007 ▶). For the synthesis of bis-1,3,4-oxadiazol, see: Al-Talib *et al.* (1990 ▶).
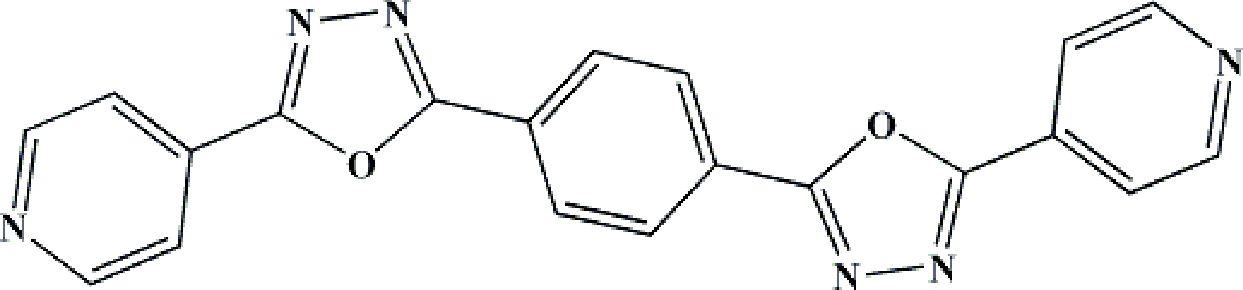


## Experimental

### 

#### Crystal data

C_20_H_12_N_6_O_2_
*M* *_r_* = 368.36
Monoclinic, 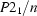

*a* = 6.2424 (6) Å
*b* = 7.6969 (7) Å
*c* = 17.7321 (16) Å
β = 96.635 (2)°
*V* = 846.27 (14) Å^3^
*Z* = 2
Mo *K*α radiation
μ = 0.10 mm^−1^
*T* = 183 K
0.30 × 0.18 × 0.15 mm

#### Data collection

Bruker SMART APEX CCD diffractometer
Absorption correction: multi-scan (*SADABS*; Sheldrick, 2003 ▶) *T*_min_ = 0.971, *T*_max_ = 0.985
4541 measured reflections
1665 independent reflections
1114 reflections with *I* > 2σ(*I*)
*R*_int_ = 0.028

#### Refinement

*R*[*F* ^2^ > 2σ(*F* ^2^)] = 0.042
*wR*(*F* ^2^) = 0.112
*S* = 1.01
1665 reflections
151 parameters
H atoms treated by a mixture of independent and constrained refinement
Δρ_max_ = 0.18 e Å^−3^
Δρ_min_ = −0.13 e Å^−3^

### 

Data collection: *SMART* (Bruker, 1999 ▶); cell refinement: *SAINT* (Bruker, 1999 ▶); data reduction: *SAINT*; program(s) used to solve structure: *SHELXS97* (Sheldrick, 2008 ▶); program(s) used to refine structure: *SHELXL97* (Sheldrick, 2008 ▶); molecular graphics: *SHELXTL* (Sheldrick, 2008 ▶); software used to prepare material for publication: *SHELXL97*.

## Supplementary Material

Crystal structure: contains datablocks I, global. DOI: 10.1107/S1600536809050557/nc2169sup1.cif

Structure factors: contains datablocks I. DOI: 10.1107/S1600536809050557/nc2169Isup2.hkl

Additional supplementary materials:  crystallographic information; 3D view; checkCIF report

## Figures and Tables

**Table 1:** Hydrogen-bond geometry (Å, °)

*D*—H⋯*A*	*D*—H	H⋯*A*	*D*⋯*A*	*D*—H⋯*A*
C3—H2⋯N3^i^	0.94 (2)	2.52 (2)	3.407 (3)	158.7 (16)

Symmetry code: (i) 
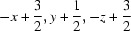
.

**Fig. 1.**
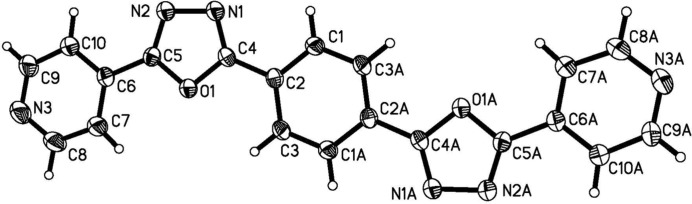
Crystal structure of the title compound showing the atom labeling scheme. Displacement ellipsoids are drawn at the 30% probability level. [symmetry code: A = x, y, z].

**Fig. 2.**
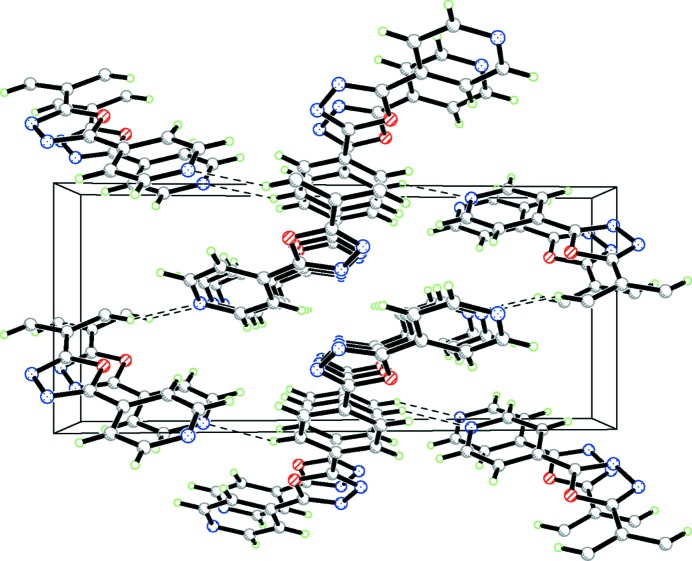
The molecular packing for the title compound along the a axis. The intermolecular C—H···N hydrogen-bonds are shown as dashed lines.

## supplementary crystallographic information

### Comment

The interest in 1, 3, 4-oxadiazole systems originate from their biological activity and their wide application in medicine, industry and coordination chemistry (Bentiss *et al.*, 2000; Navidpour *et al.*, 2006; Wang *et al.*, 2005). Substituted 1, 3, 4-oxadiazole compounds containing pyridyl group displays good coordination activities, but the related study mainly focus on mono-1,3,4 substituted oxadiazole compounds (Wang *et al.*, 2005; Zhang *et al.*, 2007). The synthesis of bis-1,3,4-oxadiazole was reported by Al-Talib *et al.*, 1990, but we have used a modified procedure. In few of the importance of oxadiazole derivatives its crystal structure is reported here.

In the crystal structure of the title compound the molecules are located on centres of inversion and are nearly coplanar. Thus, the asymmetric unit contains half a molecule (Fig. 1). In the crystal structure the molecules are stacked into columns with a centroid-centroid distances of 3.6406 (1) Å] and and 3.6287 (2) Å (Fig. 2). The columns elongate in the direction of the *a* axis and are connected via weak C-H···N hydrogen bonding (Table 1).

### Experimental

1, 4-bis[(4-pydiyl)hydrozide]phenylene (1.58 g) was added into 70 ml phosphorous oxychloride (POCl_3_) and refluxed for about 24 h. After cooling to room temperature, the mixture was poured into 500 ml water. The yellow precipitate was filtered off, washed with water, and dried. Yellow single crystals were obtained by recrystallization of the precipitate from DMF.

### Refinement

All H atoms were located in a difference Fourier map and were refined with varying coordinates and varying isotropic displacement parameters.

### Crystal data

**Table:**

C_20_H_12_N_6_O_2_	*F*(000) = 380
*M**_r_* = 368.36	*D*_x_ = 1.446 Mg m^−^^3^
Monoclinic, *P*2_1_/*n*	Mo *K*α radiation, λ = 0.71073 Å
*a* = 6.2424 (6) Å	θ = 2.3–26.1°
*b* = 7.6969 (7) Å	µ = 0.10 mm^−^^1^
*c* = 17.7321 (16) Å	*T* = 183 K
β = 96.635 (2)°	Needle, yellow
*V* = 846.27 (14) Å^3^	0.30 × 0.18 × 0.15 mm
*Z* = 2	

### Data collection

**Table:**

Bruker SMART APEX CCD diffractometer	1665 independent reflections
Radiation source: fine-focus sealed tube	1114 reflections with *I* > 2σ(*I*)
graphite	*R*_int_ = 0.028
ω scans	θ_max_ = 26.1°, θ_min_ = 2.3°
Absorption correction: multi-scan (*SADABS*; Sheldrick, 2003)	*h* = −5→7
*T*_min_ = 0.971, *T*_max_ = 0.985	*k* = −9→6
4541 measured reflections	*l* = −21→20

### Refinement

**Table:**

Refinement on *F*^2^	Primary atom site location: structure-invariant direct methods
Least-squares matrix: full	Secondary atom site location: difference Fourier map
*R*[*F*^2^ > 2σ(*F*^2^)] = 0.042	Hydrogen site location: inferred from neighbouring sites
*wR*(*F*^2^) = 0.112	H atoms treated by a mixture of independent and constrained refinement
*S* = 1.01	*w* = 1/[σ^2^(*F*_o_^2^) + (0.0489*P*)^2^ + 0.1652*P*] where *P* = (*F*_o_^2^ + 2*F*_c_^2^)/3
1665 reflections	(Δ/σ)_max_ = 0.001
151 parameters	Δρ_max_ = 0.18 e Å^−^^3^
0 restraints	Δρ_min_ = −0.13 e Å^−^^3^

### Special details

**Table:**

Geometry. All e.s.d.'s (except the e.s.d. in the dihedral angle between two l.s. planes) are estimated using the full covariance matrix. The cell e.s.d.'s are taken into account individually in the estimation of e.s.d.'s in distances, angles and torsion angles; correlations between e.s.d.'s in cell parameters are only used when they are defined by crystal symmetry. An approximate (isotropic) treatment of cell e.s.d.'s is used for estimating e.s.d.'s involving l.s. planes.
Refinement. Refinement of *F*^2^ against ALL reflections. The weighted *R*-factor *wR* and goodness of fit *S* are based on *F*^2^, conventional *R*-factors *R* are based on *F*, with *F* set to zero for negative *F*^2^. The threshold expression of *F*^2^ > σ(*F*^2^) is used only for calculating *R*-factors(gt) *etc*. and is not relevant to the choice of reflections for refinement. *R*-factors based on *F*^2^ are statistically about twice as large as those based on *F*, and *R*- factors based on ALL data will be even larger.

### Fractional atomic coordinates and isotropic or equivalent isotropic displacement parameters (Å^2^)

**Table:**

	*x*	*y*	*z*	*U*_iso_*/*U*_eq_	
O1	1.0105 (2)	0.26943 (17)	0.91503 (7)	0.0455 (4)	
N1	1.0141 (3)	0.2372 (2)	1.03852 (9)	0.0481 (5)	
N2	0.8290 (3)	0.1534 (2)	1.00208 (9)	0.0490 (5)	
C2	1.3123 (3)	0.4031 (2)	0.99232 (11)	0.0393 (5)	
C6	0.6794 (3)	0.1193 (3)	0.86751 (11)	0.0427 (5)	
C4	1.1139 (3)	0.3024 (2)	0.98550 (10)	0.0406 (5)	
C5	0.8350 (3)	0.1759 (2)	0.93029 (11)	0.0419 (5)	
C1	1.4231 (3)	0.4324 (3)	1.06365 (12)	0.0445 (5)	
C3	1.3910 (3)	0.4712 (3)	0.92850 (11)	0.0436 (5)	
N3	0.3677 (3)	0.0203 (3)	0.75019 (11)	0.0710 (6)	
C10	0.5034 (4)	0.0236 (3)	0.88153 (14)	0.0559 (6)	
C7	0.6983 (4)	0.1636 (3)	0.79320 (13)	0.0578 (6)	
C8	0.5402 (4)	0.1127 (4)	0.73749 (14)	0.0672 (7)	
C9	0.3534 (4)	−0.0212 (4)	0.82157 (14)	0.0673 (7)	
H2	1.316 (3)	0.452 (2)	0.8803 (12)	0.048 (5)*	
H1	1.365 (3)	0.390 (2)	1.1064 (12)	0.046 (6)*	
H3	0.483 (3)	−0.009 (3)	0.9316 (13)	0.063 (7)*	
H6	0.550 (4)	0.142 (3)	0.6864 (15)	0.085 (8)*	
H5	0.812 (4)	0.222 (3)	0.7800 (13)	0.069 (8)*	
H4	0.234 (4)	−0.079 (3)	0.8314 (14)	0.086 (9)*	

### Atomic displacement parameters (Å^2^)

**Table:**

	*U*^11^	*U*^22^	*U*^33^	*U*^12^	*U*^13^	*U*^23^
O1	0.0404 (8)	0.0544 (9)	0.0408 (8)	−0.0085 (6)	0.0005 (6)	−0.0010 (6)
N1	0.0434 (10)	0.0573 (11)	0.0424 (9)	−0.0076 (8)	−0.0004 (8)	−0.0024 (8)
N2	0.0453 (10)	0.0571 (11)	0.0434 (10)	−0.0091 (8)	0.0001 (8)	−0.0006 (8)
C2	0.0336 (10)	0.0411 (11)	0.0419 (11)	0.0023 (8)	−0.0017 (8)	−0.0022 (8)
C6	0.0395 (11)	0.0451 (12)	0.0425 (11)	−0.0004 (9)	0.0001 (9)	−0.0044 (9)
C4	0.0389 (11)	0.0445 (12)	0.0367 (10)	0.0018 (9)	−0.0027 (9)	−0.0033 (9)
C5	0.0362 (11)	0.0428 (11)	0.0463 (12)	−0.0027 (9)	0.0026 (9)	−0.0004 (9)
C1	0.0423 (12)	0.0521 (13)	0.0383 (11)	−0.0018 (10)	0.0012 (9)	0.0029 (10)
C3	0.0399 (12)	0.0509 (12)	0.0377 (11)	0.0003 (9)	−0.0054 (9)	−0.0013 (9)
N3	0.0657 (14)	0.0925 (16)	0.0512 (12)	−0.0132 (12)	−0.0083 (10)	−0.0080 (11)
C10	0.0573 (14)	0.0641 (15)	0.0452 (13)	−0.0159 (11)	0.0012 (11)	0.0002 (11)
C7	0.0552 (14)	0.0711 (16)	0.0464 (13)	−0.0106 (12)	0.0028 (11)	0.0003 (11)
C8	0.0718 (17)	0.0867 (19)	0.0414 (13)	−0.0059 (14)	−0.0007 (12)	0.0022 (12)
C9	0.0559 (16)	0.088 (2)	0.0559 (16)	−0.0232 (14)	−0.0020 (12)	−0.0079 (13)

### Geometric parameters (Å, °)

**Table:**

O1—C4	1.362 (2)	C1—H1	0.94 (2)
O1—C5	1.364 (2)	C3—C1^i^	1.371 (3)
N1—C4	1.288 (2)	C3—H2	0.94 (2)
N1—N2	1.413 (2)	N3—C9	1.319 (3)
N2—C5	1.290 (2)	N3—C8	1.331 (3)
C2—C3	1.387 (3)	C10—C9	1.377 (3)
C2—C1	1.388 (3)	C10—H3	0.95 (2)
C2—C4	1.454 (3)	C7—C8	1.370 (3)
C6—C10	1.369 (3)	C7—H5	0.89 (2)
C6—C7	1.379 (3)	C8—H6	0.94 (3)
C6—C5	1.457 (3)	C9—H4	0.90 (3)
C1—C3^i^	1.371 (3)		
			
C4—O1—C5	102.87 (14)	C2—C1—H1	118.9 (13)
C4—N1—N2	106.44 (15)	C1^i^—C3—C2	119.80 (18)
C5—N2—N1	105.93 (16)	C1^i^—C3—H2	120.2 (12)
C3—C2—C1	119.71 (18)	C2—C3—H2	120.0 (12)
C3—C2—C4	120.80 (17)	C9—N3—C8	116.0 (2)
C1—C2—C4	119.48 (18)	C6—C10—C9	118.9 (2)
C10—C6—C7	117.7 (2)	C6—C10—H3	120.6 (14)
C10—C6—C5	120.01 (18)	C9—C10—H3	120.5 (14)
C7—C6—C5	122.2 (2)	C8—C7—C6	119.0 (2)
N1—C4—O1	112.27 (16)	C8—C7—H5	118.8 (15)
N1—C4—C2	128.76 (17)	C6—C7—H5	122.3 (15)
O1—C4—C2	118.96 (16)	N3—C8—C7	124.1 (2)
N2—C5—O1	112.49 (16)	N3—C8—H6	115.6 (16)
N2—C5—C6	128.40 (18)	C7—C8—H6	120.3 (16)
O1—C5—C6	119.09 (17)	N3—C9—C10	124.4 (2)
C3^i^—C1—C2	120.48 (19)	N3—C9—H4	117.0 (17)
C3^i^—C1—H1	120.5 (12)	C10—C9—H4	118.5 (17)

Symmetry codes: (i) −*x*+3, −*y*+1, −*z*+2.

### Hydrogen-bond geometry (Å, °)

**Table:**

*D*—H···*A*	*D*—H	H···*A*	*D*···*A*	*D*—H···*A*
C3—H2···N3^ii^	0.94 (2)	2.52 (2)	3.407 (3)	158.7 (16)

Symmetry codes: (ii) −*x*+3/2, *y*+1/2, −*z*+3/2.
